# OxVent: Design and evaluation of a rapidly-manufactured Covid-19 ventilator

**DOI:** 10.1016/j.ebiom.2022.103868

**Published:** 2022-02-13

**Authors:** Richard Beale, Jacqueline Beddoe Rosendo, Christos Bergeles, Anair Beverly, Luigi Camporota, Alfonso A. Castrejón-Pita, Douglas C. Crockett, John N. Cronin, Timothy Denison, Sebastian East, Chantal Edwardes, Andrew D. Farmery, Filiberto Fele, James Fisk, Carla V. Fuenteslópez, Michael Garstka, Paul Goulart, Clare Heaysman, Azad Hussain, Prashant Jha, Idris Kempf, Adhithya Senthil Kumar, Annika Möslein, Andrew C.J. Orr, Sebastien Ourselin, David Salisbury, Carlo Seneci, Robert Staruch, Harrison Steel, Mark Thompson, Minh C. Tran, Valentina Vitiello, Miguel Xochicale, Feibiao Zhou, Federico Formenti, Thomas Kirk

**Affiliations:** a Centre for Human and Applied Physiological Sciences, King's College London, UK; b Intensive Care Unit, Guy's and St Thomas' NHS Foundation Trust, London, UK; c School of Biomedical Engineering and Imaging Sciences, King's College London, UK; d Department of Engineering Science, University of Oxford, UK; e Nuffield Department of Clinical Neurosciences, University of Oxford, UK; f Milton Keynes University Hospital NHS Foundation Trust, Milton Keynes, UK; g Department of Anaesthesia, Guy's and St Thomas’ NHS Foundation Trust, London, UK; h Department of Engineering Science, Institute of Biomedical Engineering, University of Oxford, UK; i Medical Sciences Division, University of Oxford, UK; j Department of Biomechanics, University of Nebraska Omaha, Omaha, NE, USA; k OxVent Ltd, Abingdon, UK; l Nuffield Department of Orthopaedic, Rheumatology and Musculoskeletal Sciences, University of Oxford, UK; m The Academic Department of Military Surgery and Trauma, Birmingham, UK

**Keywords:** Respiration (artificial), Biomedical engineering, Covid-19, Critical care, ASV, assisted spontaneous ventilation, CO, cardiac output, CRS, respiratory system compliance, DBP, diastolic blood pressure, F_I_O_2_, Fraction of inspired oxygen, Hb, haemoglobin, HR, heart rate, LoA, limits of agreement, MAP, mean arterial pressure, PaCO_2_, arterial partial pressure of carbon dioxide, PADP, pulmonary artery diastolic pressure, PaO_2_, arterial partial pressure of oxygen, PASP, pulmonary artery systolic pressure, PEEP, positive end-expiratory pressure, PIP, proportional integral derivative, PFR, PaO_2_: F_I_O_2_ ratio, RAW, airways resistance, SaO_2_, arterial oxygen saturation, SBP, systolic blood pressure, Texp, expiratory time, Tinsp, inspiratory time, VCV, volume controlled ventilation, VT, tidal volume

## Abstract

**Background:**

The manufacturing of any standard mechanical ventilator cannot rapidly be upscaled to several thousand units per week, largely due to supply chain limitations. The aim of this study was to design, verify and perform a pre-clinical evaluation of a mechanical ventilator based on components not required for standard ventilators, and that met the specifications provided by the Medicines and Healthcare Products Regulatory Agency (MHRA) for rapidly-manufactured ventilator systems (RMVS).

**Methods:**

The design utilises closed-loop negative feedback control, with real-time monitoring and alarms. Using a standard test lung, we determined the difference between delivered and target tidal volume (VT) at respiratory rates between 20 and 29 breaths per minute, and the ventilator's ability to deliver consistent VT during continuous operation for >14 days (RMVS specification). Additionally, four anaesthetised domestic pigs (3 male-1 female) were studied before and after lung injury to provide evidence of the ventilator's functionality, and ability to support spontaneous breathing.

**Findings:**

Continuous operation lasted 23 days, when the greatest difference between delivered and target VT was 10% at inspiratory flow rates *>*825 mL/s. In the pre-clinical evaluation, the VT difference was -1 (-90 to 88) mL [mean (LoA)], and positive end-expiratory pressure (PEEP) difference was -2 (-8 to 4) cmH_2_O. VT delivery being triggered by pressures below PEEP demonstrated spontaneous ventilation support.

**Interpretation:**

The mechanical ventilator presented meets the MHRA therapy standards for RMVS and, being based on largely available components, can be manufactured at scale.

**Funding:**

Work supported by Wellcome/EPSRC Centre for Medical Engineering,King’s Together Fund and Oxford University.

**Research in context**

**Evidence before this study**

The Covid-19 infection started spreading in the UK in March 2020. Based in part on the impact of high infection rates on national health services in Italy earlier in March 2020, the UK Government expected a rapid increase in the number of admissions to intensive care, and an associated requirement for greater mechanical ventilation capacity. In response to this predicted need, and considering the long time needed to manufacture standard mechanical ventilators (due to their complexity and to the limited availability of their components), the UK Government instituted the Ventilator Challenge, aimed at upscaling the manufacture of ventilators, including safe, novel designs that could be manufactured rapidly. In parallel, the Medicines and Healthcare products Regulatory Agency (MHRA) published the technical specifications that defined the minimum clinical standards acceptable for ventilators to be used in UK hospitals during the Covid-19 outbreak.

The OxVent mechanical ventilator project was submitted to the Challenge and was selected as one of the 16 projects (out of 5,000 submissions) to further develop and prepare for manufacture.

**Added value of this study**

To the best of our knowledge, the OxVent mechanical ventilator is the only design submitted to the Challenge that was able to scale to manufacturing thousands of units per week, within a matter of weeks. It provides a relatively advanced ventilation support in the form of assisted spontaneous ventilation, and the cost of its materials is limited to ∼ £1,000. This study presents an overview of the OxVent's design and the results from internal verification and pre-clinical testing to demonstrate its functionality. The ventilator operated continuously for more than 3 weeks, can provide therapy for much of the operating envelope set out in the UK rapidly manufactured ventilator systems’ specifications, and its functionality was robust in a simulated emergency context where air supply pressure was varied around the nominal design point.

**Implications of all available evidence**

The actual need for mechanical ventilation capacity in the UK during 2020–21 was a fraction of that predicted in March 2020, allowing the National Health Service to adopt the lower-risk strategy of using machines that were all adaptations of existing approved products. As such the OxVent was not manufactured at scale. This situation ultimately avoided the potential risks associated with the use of novel ventilators from manufacturers without an established track record, while providing extra mechanical ventilation capacity, should it have been needed.

Though the concept of open-source designs for medical devices was very appealing during the early stages of the pandemic, it poses numerous challenges for translation to clinical settings: any medical device design needs thorough testing, verification, validation, and to be manufactured under a certified Quality Management System. Only in the ultimate stage of this extensive process, a long way after the open-source design stage, can a device achieve regulatory approval and be used in hospitals.

The work presented here was the basis for the establishment of OxVent Ltd. By obtaining approval for a production unit in the UK and then transferring the designs and manufacturing systems to organisations in low and middle-income countries (LMICs), OxVent aims at bridging the gap between the mechanical ventilator verification stage and its adoption in hospitals in LMICs, where expensive ventilators are unaffordable and unsustainable.

Alt-text: Unlabelled box

## Introduction

### Background

Early during the Covid-19 pandemic, invasive mechanical ventilation was employed extensively to treat respiratory failure.^1^ In response to an anticipated shortage in intensive care ventilation capacity due to exceptional demand, the UK Government launched the *Ventilator Challenge*, seeking both conventional and novel designs of ventilator systems that could be manufactured at scale within a few weeks. The requirements for such systems were provided by the Medicines and Healthcare products Regulatory Agency (MHRA) in the rapidly manufactured ventilator systems (RMVS) specification document of March 2020^2^ and three updates to April 2020, the key points of which are presented in Table 1. In the US, the Food and Drug Administration (FDA) set out requirements for the emergency use authorisation of devices,^3^ and in Mexico, COFEPRIS released specifications similar to the RMVS.^4^ At the time of the challenge being launched, there were chronic shortages affecting almost all medical supply chains (by April 2020, 69 countries had imposed export restrictions on medical products or food).^5^ This shortage provided an important justification for seeking novel designs, which could be manufactured *outside* of impacted supply chains.

**Table 1:** Key requirements from the RMVS specification. Trigger pressure pertains to assisted spontaneous ventilation only.

*Parameter*	*Limits*
Tidal volume (VT)	250–600 mL
Respiratory rate (RR)	10–30 / min
Inspiratory-to-expiratory time (I:E) ratio	2:1 - 1:3
Inspiratory pressure limit (Plimit)	15–40 cmH_2_O
Positive end-expiratory pressure (PEEP)	5–20 cmH_2_O
Trigger pressure [below PEEP]	1–10 cmH_2_O
Fraction of inspired oxygen (F_I_O_2_)	30–100%

In response to the Ventilator Challenge, the OxVent ventilator was designed by a team of clinicians and engineers at the University of Oxford and King's College London. The system was one of 16 candidate devices (out of 5,000) selected by the UK Government for further development, and an initial order for 6,000 units was placed in April 2020. Concurrently, Smith and Nephew joined the project to further develop and manufacture the device at scale; based on the concept prototype originally supported by the UK Ventilator Challenge, they designed the final ventilator presented here and, most critically, setup a manufacturing and testing line that uniquely enabled the scalability to 5,000 ventilators per week (equivalent to ∼ 30 ventilators per hour on a 24 h, 7 days per week basis). We present here an overview of the OxVent's design, verification testing against the RMVS specification, evidence of its functionality in a porcine model (with and without acute lung injury), and demonstrate the ventilator's assisted spontaneous ventilation capability.

### System design

**Figure 1:**
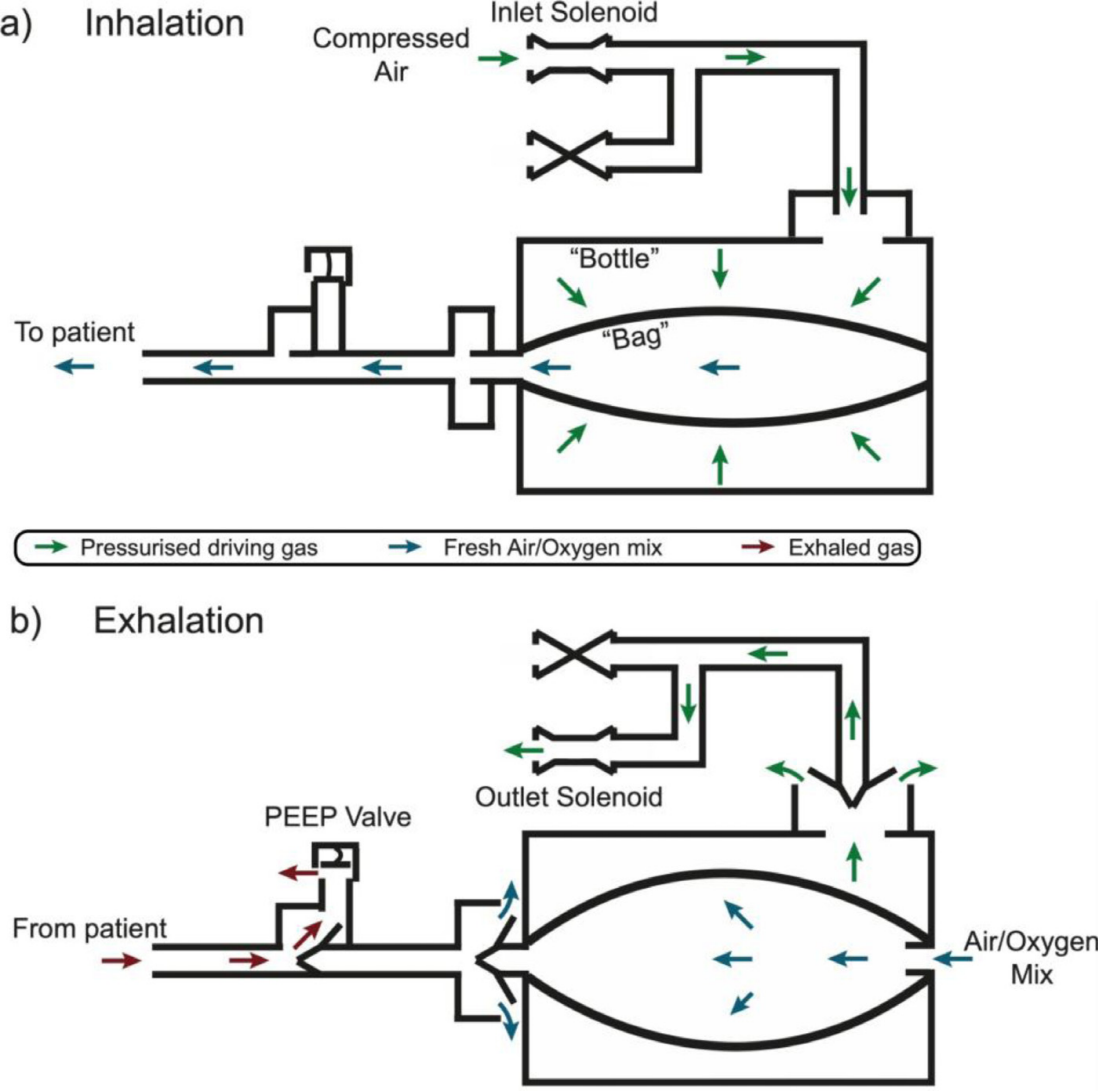
Overview of ‘bag in bottle’ principle. During inhalation (a), the resuscitator bag is compressed within a sealed pressure vessel (‘bottle’). During exhalation (b), patient air exits through the PEEP valve; the bottle exhausts through a separate solenoid. Note: heat and moisture exchange (HME) filters are not shown for clarity.

The OxVent is a ‘bag in bottle’ device. A single-use resuscitator bag [Visionary, Marshall Products, UK] is enclosed within a sealed chamber (illustrated in Figure 1). Using an external compressed air supply, the chamber is pressurised, causing the bag to expel air into the patient airway via standard breathing tubing, inspiratory valve, and heat and moisture exchange filters. Because there is complete separation between the compressed air that is used to drive the system and that which is delivered to the patient, the system may be run using an industrial compressed air supply (as opposed to purified medical air).

Two modes of ventilation are provided: volume-controlled (VCV) and Assisted Spontaneous Ventilation (ASV). The former mandatory mode delivers a target VT at a set RR and I:E ratio, whereas the latter is triggered by inspiratory effort and can therefore be used to support weaning. Ventilation under VCV can be provided according to the parameters given in Table 1. Under ASV, ventilation is triggered by a drop in airway pressure below PEEP, indicating the patient attempted inspiratory effort (the airway pressure drop threshold may be set from -1 to -10 cmH_2_O). PEEP is difficult to measure, both due to inter-breath variability and inherent sensor noise. A windowing approach that discards sensor readings during the first 75% of expiration was used for both VCV and ACV to determine a PEEP value within the current ventilatory cycle: at the minimum sampling rate of 25 Hz and expiration time of 1 s, this approach ensures at least 5 sensor readings were used to calculate PEEP. For ACV only, it was further necessary to calculate ‘baseline’ PEEP, which is a moving average of PEEP over cycles, and below which a pressure drop triggers inspiration. This baseline was calculated using the following auto-regressive relation: $PEEP_{baseline}\left(i+1\right)=\beta PEEP_{baseline}\left(i\right)+\left(1-\beta \right)PEEP\left(i\right)$ where the next (i+1) baseline value was calculated from the baseline and PEEP value for the current (i) cycle. The weighting parameter β balanced noise rejection against responsiveness to short-term changes (for example, an adjustment of the PEEP valve itself); following experimentation, a β value of 0.7 was deemed appropriate. Under both modes of ventilation, an alarm sounds if the user-set maximum inspiratory pressure (Plimit) threshold is met, though both software and hardware controls will prevent the threshold pressure from being exceeded. An external oxygen supply connects directly to the resuscitator bag to control the fraction of inspired oxygen (F_I_O_2_) in the patient airway via rotameter (which is monitored in real-time by an internal sensor). At nominal settings of VT = 400 mL, RR = 20/min and F_I_O_2_ = 50%, oxygen consumption is 4 L/min, well within the upper bound of 6 L/min set out in the RMVS specification.^2^ This oxygen consumption is lower than that of comparable transport or emergency devices^6^^,^^7^; is 2, 3 times smaller than that of a standard ICU ventilator,^8^^,^^9^ and compares favourably with continuous positive-airway pressure systems.^10^

**Figure 2:**
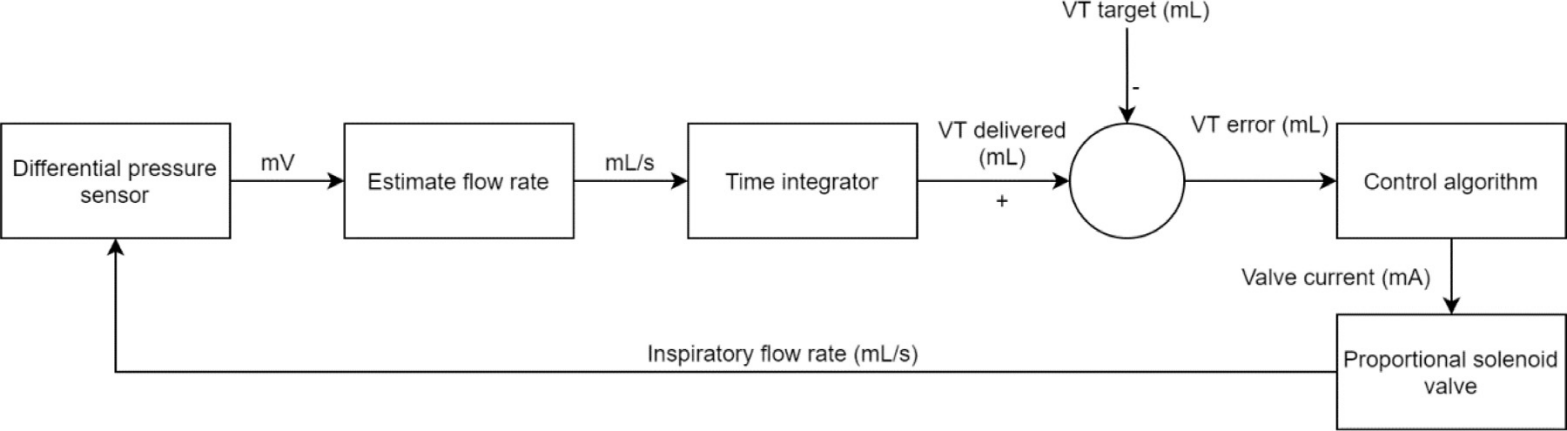
System block diagram for the closed-loop negative feedback proportional, integral, derivative (PID) algorithm used within the system. The control algorithm adjusts the solenoid valve current based on the error between the integrated tidal volume (VT) delivered and the set target volume; this VT error is calculated on a breath-by-breath basis.

System control, the schematic of which is illustrated in Figure 2, is implemented via a closed-loop negative feedback proportional, integral, derivative (PID) algorithm running on an Arduino Nano 33 BLE development board [Arduino, Italy]. PID control is well-established for continuous systems and minimises steady-state error, rejects constant disturbances, and is robust to high-frequency sensor noise.^11^ Particularly for sustained use over a period of days, closed-loop control offers substantial safety advantages over some of the alternative open-loop designs submitted to the Ventilator Challenge. This is especially beneficial in light of the non-linearity of pneumatic systems, which will magnify any errors in the output of an open-loop controller. The OxVent system requires only a calibration of the pressure/flow sensor, and not, crucially, knowledge of the precise relationship between the current provided to the solenoid valve [PVQ31-5G-16-01F, normally closed type, SMC Pneumatics, USA] and the flow that results (which can vary between individual examples). The controller has been deliberately overdamped to reduce the possibility of volume overshoot; the penalty for this is a longer response time to step changes in requested therapy (usually around 10 cycles). The relatively high capability of the 32-bit ARM Cortex-M4 CPU on the Arduino board allows for functionality to be implemented in firmware instead of hardware. For example, when the RMVS specifications were changed to require ASV functionality, some weeks after the start of the Ventilator Challenge, this implementation was achieved via a firmware update, and no hardware changes were required. This approach represents a considerable saving in design effort and reduces the time required to modify the system in light of changing needs and specifications.

**Figure 3:**
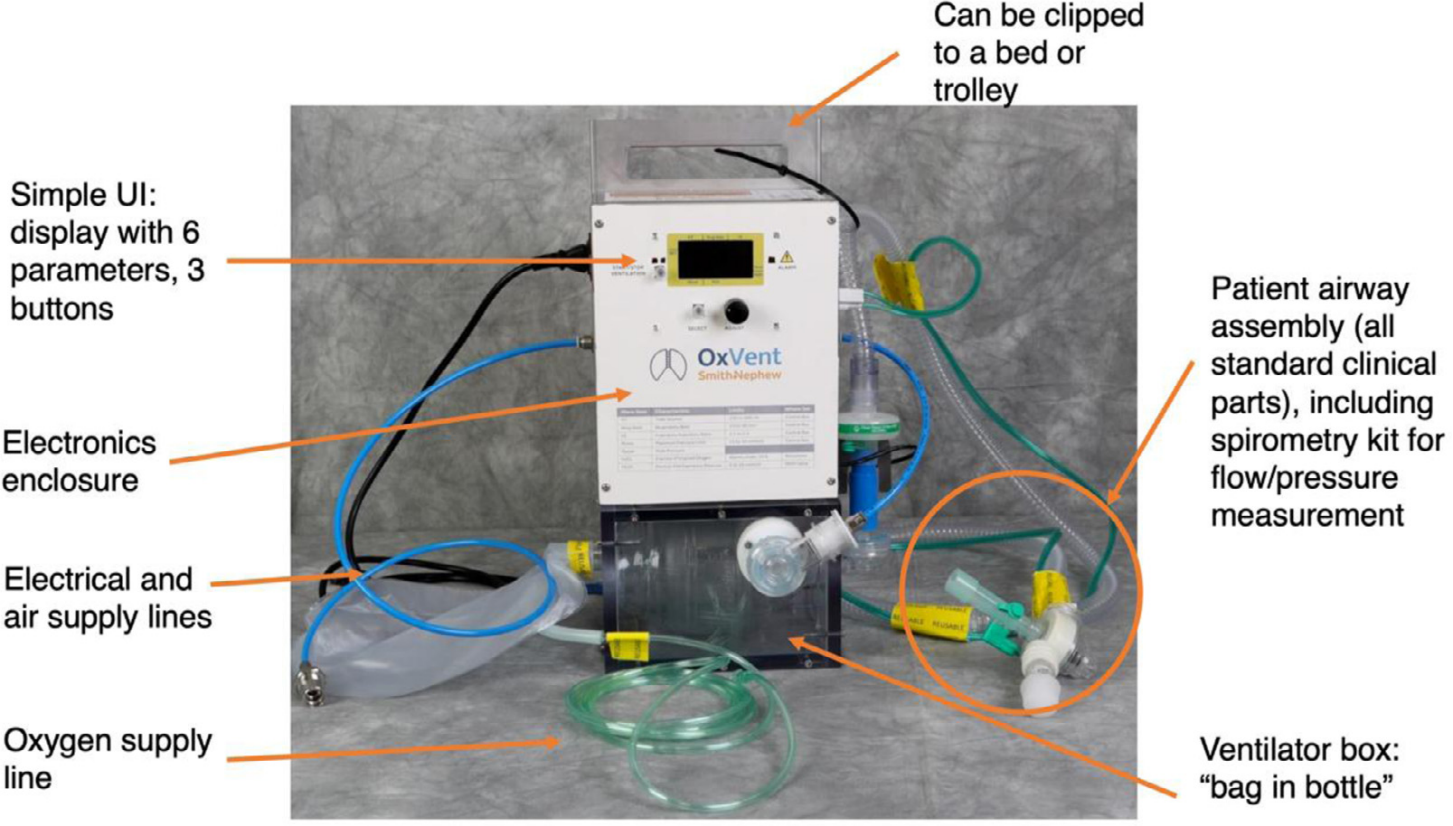
The OxVent system, with all required supply and patient connections. The device comprises two main assemblies: the electronics/control enclosure (white panel, top), and ventilator box (‘bag in bottle’, bottom). The system has inlets for electrical power, compressed air at 4 bar, and oxygen (the concentration of which is set via rotameter). Flow measurement is achieved via a spirometry kit placed close to the patient airway.

The physical form of the OxVent (shown in Figure 3) has been optimised for rapid manufacture and durability. It is 480 mm high, 290 mm wide and 240 mm deep, weighs 7.25 kg, and can stand on the floor or be clipped to a bed or trolley. The body consists of a single sheet of laser-cut and folded steel, onto which the ventilator box assembly and control panel are bolted. The majority of electrical components are integrated directly onto the main circuit board at the time of manufacture of this component, which minimises the amount of assembly required further down the line. As part of the design for manufacture process undertaken by Smith & Nephew, the device passed electromagnetic compatibility and electrical safety tests performed in April 2020.

All patient-facing components (resuscitator bag, heat and moisture exchange (HME) filter, smoothbore hose, spirometry kit, pressure release valve and PEEP valve) are inexpensive single-use parts currently in widespread clinical use. This solution ensures biological safety, familiarity for users and, by replacing these parts between patients, helps minimise the risk of cross-contamination. The spirometry kit is positioned as close to the patient airway as possible to minimise the impact of instrumental deadspace upon flow measurement.

The OxVent uses widely-available electronics to provide real-time monitoring and control of ventilation. Standard and commonly available patient-facing components such as the resuscitator bag and patient airway assembly are sourced from clinical supply chains, while all electronics are sourced elsewhere (for example, the medical grade oxygen sensor is popular for scuba diving). Such parallel sourcing was crucial in avoiding the global supply bottlenecks that existed during the early stages of the pandemic and enabled production capacity to be rapidly scaled.

## Methods

### Design verification testing

Design verification testing against the RMVS specification was performed in VCV mode using three identical test lungs of fixed resistance 20 cmH_2_O/(L/s) and compliance 17.7 mL/cmH_2_O (values consistent with ARDS) [SmartLung Adult, IMT Analytics AG, Switzerland]. Reference tidal volume measurements were taken using an IMT FlowAnalyser PF-3000 [IMT Analytics AG, Switzerland]. In total, 14 separate tests were performed to investigate various aspects of ventilation capability (the full dataset and results may be accessed at)^12^; since most of these tests were under nominal conditions, three tests that investigated the limits of ventilation capability are reported here. Where VT measurements were taken over consecutive breathing cycles, violin plots are used to convey the distribution of individual values. Numerical analysis was performed with Python 3.8.5, Numpy 1.20.3 and Pandas 1.3.2 [www.python.org, www.numpy.org, www.pandas.pydata.org].

### Maximum achievable therapy

The bag in bottle design of the OxVent intrinsically limits the maximum VT that can be supplied. This is due the finite volume of the resuscitator bag (1 L) and the finite rate at which it can be compressed. Hence, a few high-flow rate configurations included in RMVS specifications may be outside of the capability of the device, for example VT = 600 mL, RR = 30/min, and I:E ratio = 1:3 which corresponds to a flow rate of 1200 mL/s. Three OxVent devices were run at various high-flow rate configurations and VT delivered was averaged over 10 consecutive cycles. Due to the very large number of possible therapy configurations included within the RMVS specifications, only certain configurations with flow rates between 750 and 900 mL/s were tested as it was observed during preliminary work that the limits of system capability were in this range. Delivered VT was recorded and the lowest result amongst the three devices reported to give a lower bound on performance. For all tests, Plimit was set at 45 cmH2O to avoid pressure-limiting behaviour. Note that this experimental setup only investigated under-delivery of VT (arising due to the physical limitations of the bag in the bottle design), whereas over-delivery, if observed, is not an inherent limitation of the bag in bottle design and should be dealt with by the controller.

### Variation in supply pressure

The OxVent requires an external 4 bar compressed air supply. In a crisis situation, during which hospital infrastructure is overwhelmed, it is possible that units may need to operate with air supply pressure away from the nominal design point. This could be the case if too many units were connected to a single source, for example. To investigate performance under such conditions, a unit was set to operate at VT = 600 mL, RR = 25/min, I:E ratio = 1:2 at pressures of 4.0, 3.5 and 3.0 bar. Separately, a unit was set to operate at VT = 450 mL, RR = 15/min, I:E ratio = 1:2 at pressures of 4.0, 4.5, 5.0, 5.5 and 6.0 bar. In both cases, VT delivered was recorded for 10 consecutive cycles once steady state was reached.

### Long-term ventilation

The RMVS specifies a system lifetime of at least 14 days of uninterrupted operation. The OxVent makes use of numerous relatively inexpensive and disposable parts designed for temporary therapeutic use, most notably the resuscitator bag and associated valves, and as such the complete system does not have an indefinite lifetime (though the modular design makes it possible to replace certain worn-out parts). To investigate system lifetime under high-flow rates, a single unit was set to operate at VT = 600 mL, RR = 27/min, I:E ratio = 1:2, PEEP = 10 cmH_2_O and Plimit = 45 cmH_2_O until failure (defined as delivered VT > ± 10% deviation from target). VT delivered was recorded for 10 consecutive cycles every 24 h.

### Pre-clinical testing

Four pigs (3 male, 1 female; *Sus scrofa* domesticus) were studied under general anaesthesia with rocuronium for muscle relaxation, before and after saline-lavage lung injury^13^ to demonstrate the ventilator's functionality also in the context of limited pulmonary compliance.^14^ The rocuronium was stopped and sugammadex administered (to reverse neuromuscular blockade) for the ASV part of the experiment, lasting approximately 30 min. Further details of the technique are presented elsewhere.^15^ Vital signs were monitored throughout [multiparameter monitor: Datex, AS3, Finland; respiratory monitor: Datex Ohmeda [Capnomac Ultima, Finland], and end-expiratory lung volume was measured using SF6-washout.^16^ The anaesthetised animals were euthanised with a bolus dose of potassium chloride (1, 2 mmoL kg^−1^) upon completion of the study protocol.

Two prototype ventilators were operated by two users, one with no mechanical ventilation experience to test usability. Animals were ventilated at increasing positive end-expiratory pressure (PEEP) levels (5, 10 and 20 cmH_2_O) with clinically relevant tidal volume (VT) of 6 and 11 mL/kg, inspired-to-expired ratio (I:E) of 1:2 and 2:1, and respiratory rates of 28 and 16 breaths/min [OxVent, or Servo-I, Maquet Critical Care, Solna, Sweden]. Due to the limited weight (∼ 30 kg) of the pigs studied, the absolute VT was limited to a maximum of c. 330 mL. OxVent's delivery of set ventilatory parameters was compared against those measured by spirometry. Analogue signals were continuously recorded on a computer via PowerLab and LabChart [AD Instruments, New Zealand].

### Statistics

Agreement between parameters set on the OxVent mechanical ventilator and those measured by simultaneous spirometry was assessed with Bland-Altman analysis.^17^ Data were processed using R version 3.6.2 (www.r-project.org). Values presented are mean ± SD unless otherwise stated.

### Independent evaluation

As part of the Ventilator Challenge, an independent evaluation of the system against RMVS specifications was performed by the Medical Devices Testing and Evaluation Centre [Birmingham, UK] in April 2020. The full text of their report may be accessed at.^18^ Qualitative findings from this evaluation will be highlighted in the discussion section.

### Ethics

Pre-clinical testing conformed to the Animal Research: Reporting of *in vivo* Experiments guidelines,^19^ and ethical approval was granted by the Uppsala Regional Ethics Committee (ref. C98/16).

### Role of funders

The funders had no role in the study and the writing of this article.

## Results

### Design verification testing

**Figure 4:**
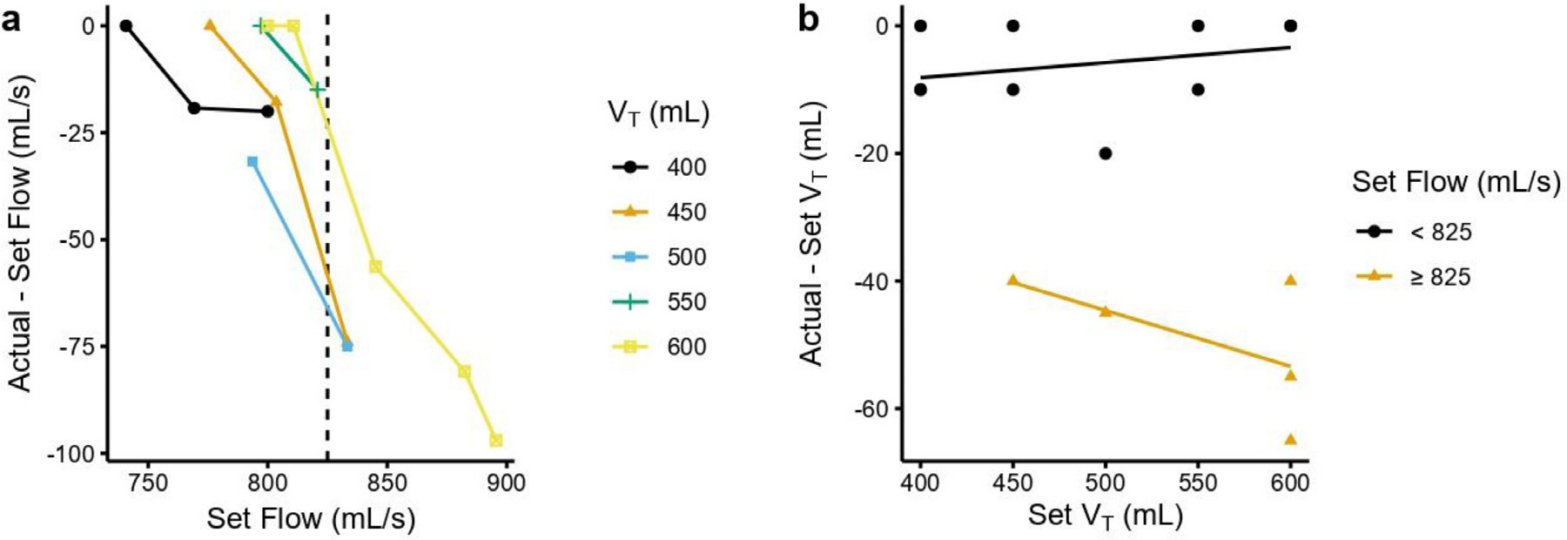
Limits of VT delivery in different flow rates configurations. In each case, the worst-case result amongst three test devices is shown, measured as the difference between delivery and set value. a) VT delivery error versus set inspiratory flow rate showed greater under-delivery for flows >825 mL/s. b) Re-plotting the same data as a function of set VT shows that high VTs could be attained with flow rates under 825 mL/s, whereas even lower VTs were unattainable with flows at or above 825 mL/s (for example, 40 mL under-delivery at VT = 450 mL and flow >825 mL/s).

Figure 4 shows the results of the maximum achievable therapy test. Across all combinations of VT, RR and I:E ratio, the extent of VT under-delivery (difference between actual and set VT) increased as inspiratory flow rate increased (panel a). This difference was particularly notable for flows above 825 mL/s. Panel b demonstrates that VT in isolation was not predictive of under-delivery: whilst large VTs could readily be attained with lower flow rates, even smaller VTs were challenging once required flow was above 825 mL/s. It follows that this flow rate is close to the limit of what the resuscitator bag can supply under rapid and complete compression.

**Figure 5:**
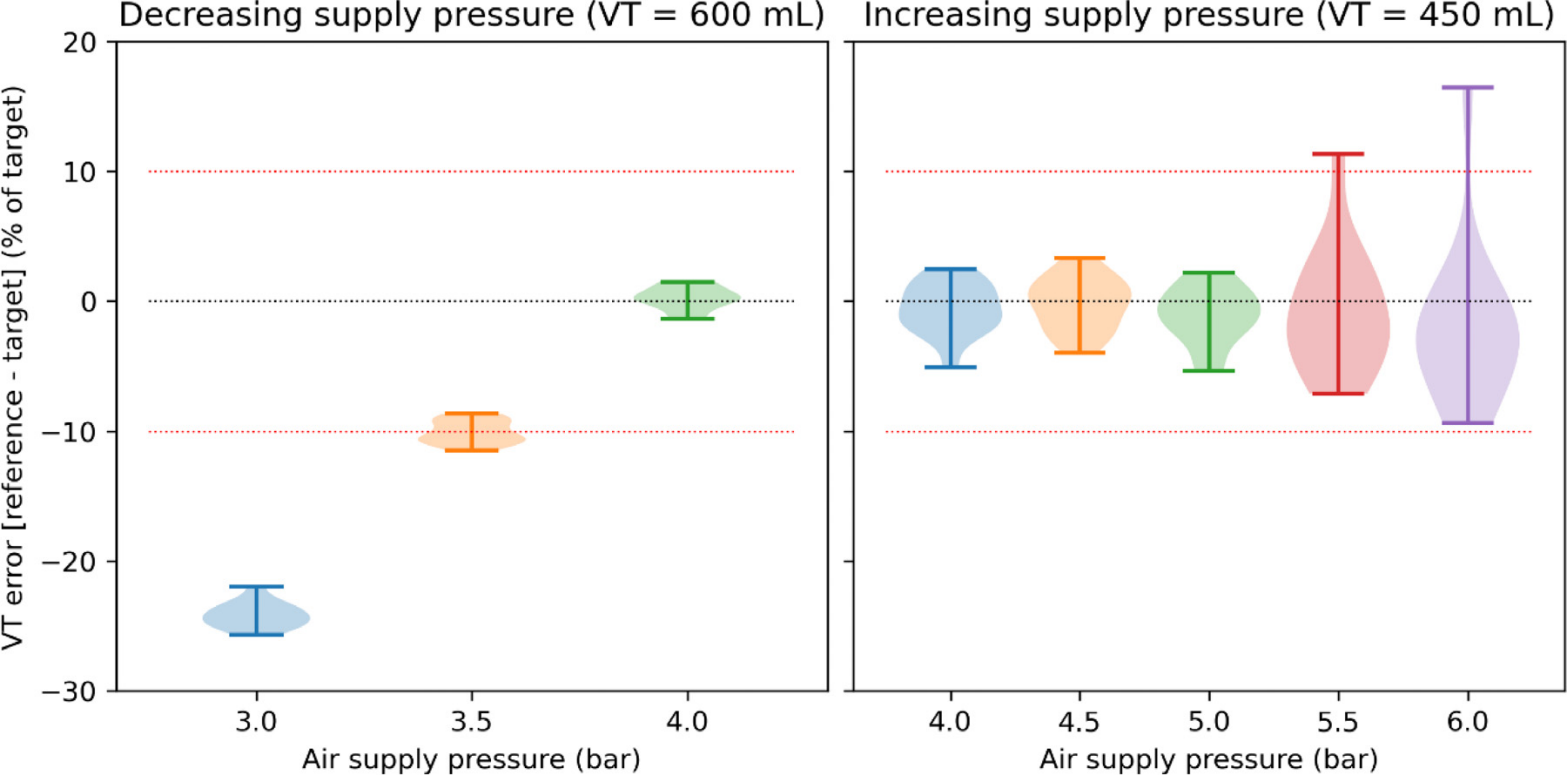
Variation in supply pressure, both decreasing (left) and increasing (right). Violin plots over consecutive cycles are shown, and error bounds of ± 10% are denoted with red dotted lines. Different colours illustrate the different supply pressure conditions tested. For decreasing pressures, mean VT delivery was within 10% of target at and above 3.5 bar (delivery at 3.5 bar was right on the lower bound). For increasing pressures, mean VT delivery was robust at all pressures up to and including 6 bar, though inter-breath variability exceeded the bounds at higher pressures.

Figure 5 shows the results of the variation in supply pressure test. For decreasing pressures, VT delivery was within a ±10% error bound for pressures above 3.5 bar. For increasing pressures, mean VT delivery was within these same bounds for pressures up to 6 bar, though inter-breath variability exceeded the bounds at the highest pressures.

**Figure 6:**
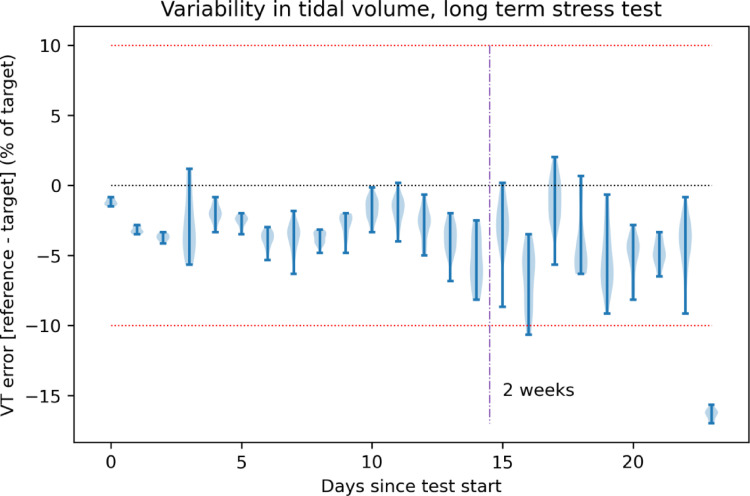
Long-term ventilation under high-flow rates (VT = 600 mL, RR = 27/min, I:E = 1:2). Violin plots over consecutive cycles are shown, and error bounds of *±*10% are denoted with red dotted lines. VT delivery was within bounds for 23 days (surpassing the two-week RMVS requirement); the test was terminated at 24 days due to mechanical failure of the resuscitator bag after approximately 860 k cycles.

Figure 6 shows the results of the long-term test. VT delivery was within the error bounds of ± 10% around target for 23 days; the test was terminated due to mechanical failure of the resuscitator bag (gradual degradation of the silicone vanes that prevent return flow within the outlet valve) after 24 days.

### Pre-clinical testing

Table 2 summarises the animals’ median baseline characteristic, showing a 66% lower PaO_2_:F_I_O_2_, 52% lower end-expiratory lung volume, and 59% lower respiratory system compliance after lung injury.

**Table 2:** Baseline parameters before and after lung injury in four anaesthetised pigs. Measurements were recorded at PEEP = 5 cmH_2_O, and demonstrate the reduced pulmonary gas exchange and compliance associated with lung injury. Values are median (LoA). Abbreviations are presented at the end of the article.

*Parameter*	*Pre-injury*	*Post-injury*
Weight (kg)	30.7 (29.9–31.1)	
MAP (mmHg)	72 (66–80)	88 (87–88)
CO (L/min)	3.47 (2.89–3.82)	3.16 (2.46–3.76)
PASP (mmHg)	24 (20–28)	32 (31–34)
PADP (mmHg)	12 (9–14)	19 (15–21)
F_I_O_2_	0.30 (0.29–0.31)	0.67 (0.61–0.71)
SaO_2_ (%)	99 (99–99)	99 (98–100)
pH	7.48 (7.46–7.49)	7.32 (7.24–7.37)
PaO_2_(kPa)	18.4 (17.5–20.3)	32.0 (26.4–33.5)
PaCO_2_(kPa)	4.9 (4.7–5.1)	7.1 (6.5–8.4)
PFR (mmHg)	446 (429–456)	294 (201–392)
End-Expiratory Lung Volume (mL)	512 (462–621)	265 (223–314)
CRS (mL/cmH_2_O)	22 (20–23)	13 (11–16)
Raw (cmH_2_O/(L/s))	7.5 (6.3–8.7)	7.4 (5.6–10.8)

Table 3 shows the comparison between set and measured ventilatory parameters; VT limits of agreement (-90 - +88 mL) were 50% of a 6 mL/kg breath for a 30 kg animal. Inspiratory time was up to 0.48 s less than desired, associated with a proportionally longer expiration.

**Table 3:** Bland-Altman analysis comparing the parameters set on the OxVent against those measured by simultaneous spirometry.

*Parameter*	*Mean Difference*	*95% Limits of Agreement*
VT (mL)	-1	-90 to 88
PEEP (cmH_2_O)	-2	-8 to 4
Tinsp (ms)	-307	-480 to -133
Texp (ms)	390	217 to 565

**Figure 7:**
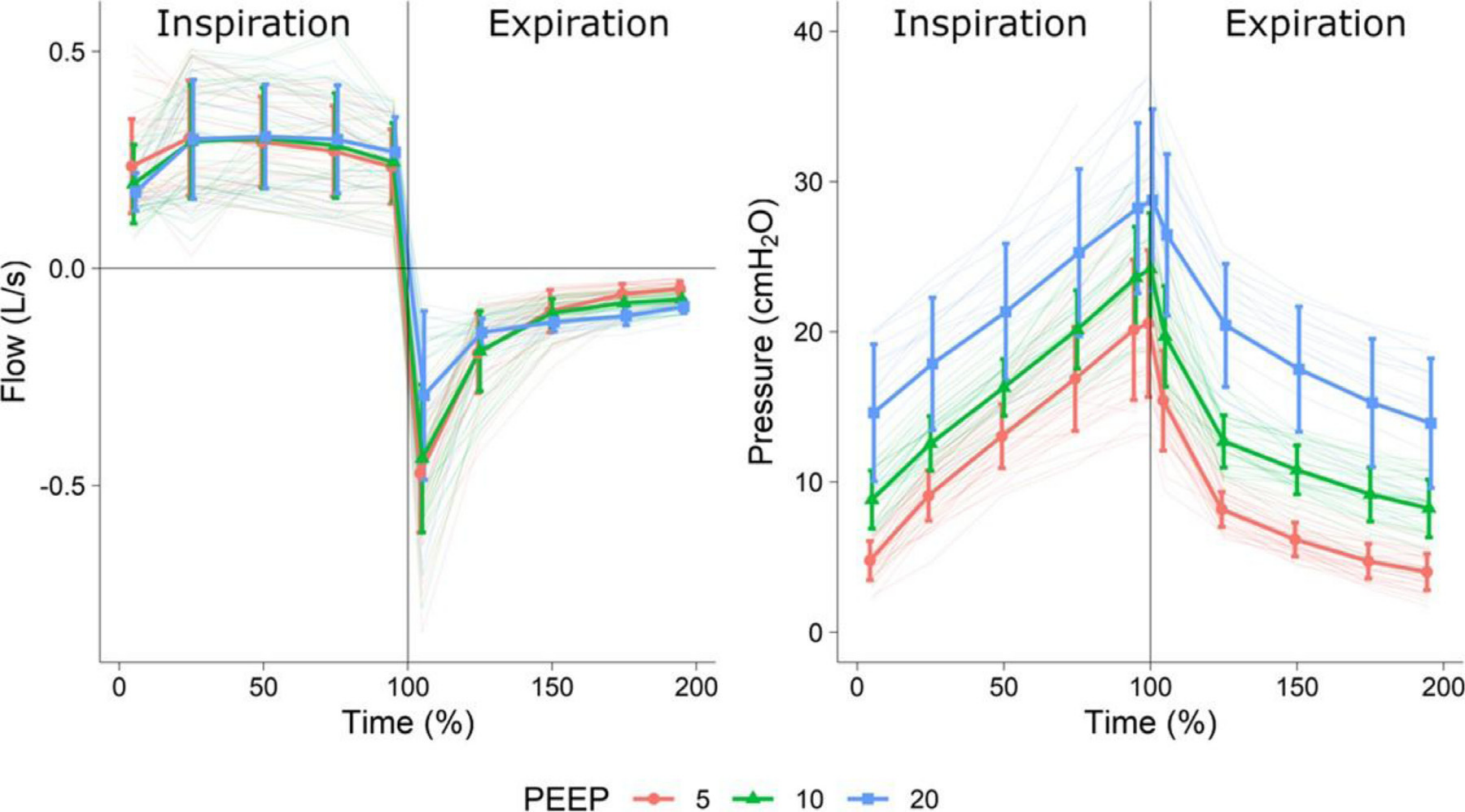
Flow and pressure characteristics. To facilitate the comparison between conditions, time is provided as percentage of inspiration/expiration due to different inspiratory/expiratory times used during the protocol. Thin lines represent each experiment, while thick lines show values grouped by PEEP [points and error bars represent mean (SD)]. Different colours represent different levels of PEEP in cmH_2_O: PEEP 5 [red; *n* = 35], PEEP 10 [green; *n* = 32], PEEP 20 [blue; *n* = 17].

Figure 7 shows the profile of flow and pressure delivered over the course of an expiration. PEEP was not maintained, particularly at the highest PEEP level tested of 20 cmH_2_O.

**Figure 8:**
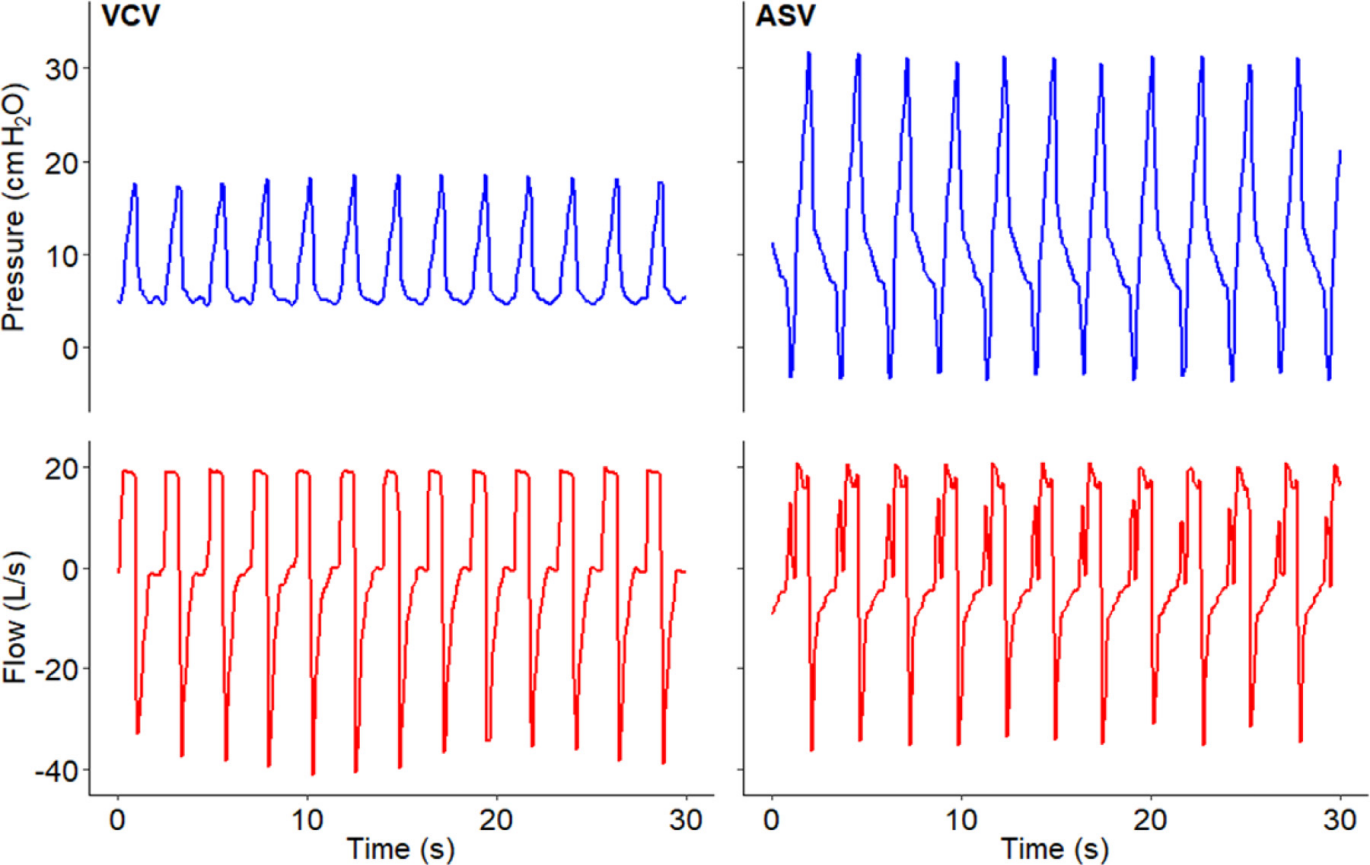
Example 30 s periods of ventilation showing volume-controlled mandatory ventilation (VCV) in the left panel and assisted spontaneous ventilation (ASV) in the right panel [*n* = 1]. ASV was tested after lung injury, when pulmonary compliance was reduced, hence leading to greater peak inspiratory pressures.

Figure 8 shows example periods of mandatory (VCV) and assisted spontaneous ventilation (ASV) recorded during the study, illustrating the sub-PEEP levels that triggered the delivery of breaths in the latter condition. PEEP was set at 5 cmH_2_O in each test to facilitate the comparison between the conditions, where volume-controlled ventilation was tested in pigs with uninjured lungs, and assisted spontaneous ventilation was tested in pigs after lung injury with saline lavage.

## Discussion

### Design verification testing

The OxVent successfully delivered a subset of clinically relevant ventilation settings, required by the RMVS specifications (including the nominal defaults set out in the specifications). Combinations of large VT, high RR, and high I:E ratio, which together imply inspiratory flow rates in excess of 825 mL/s, resulted in a >10% reduction in delivered VT. Although it is debatable whether such configurations would ever be required in a therapeutic setting, the system's software could be updated to prevent users from requesting unattainable settings.

Ventilation was robust in the presence of variation in air supply pressure. This finding is encouraging given that the system is designed for use in a crisis situation, during which hospital infrastructure may be overwhelmed and therefore unable to provide nominal air supply pressure. This may also facilitate the use of the ventilator in limited resource settings, as long as compressed air is available.

Finally, the system continuously operated under high-flow rate for 23 days, substantially exceeding the 14 day minimum lifetime required by the MHRA RMVS. Given that many patient-facing components of the ventilator are designed for temporary use, the failure mode of this test (failure of the resuscitator bag) is not entirely unexpected. However, given the modular design of the ventilator, all such parts can be replaced to extend the operating lifetime. Improvements in design may include a self-test of the resuscitator bag on power-up to confirm the ventilator can operate as expected, or to alert the user on the need to replace the resuscitator bag, and a quicker setup for its replacement. It would also be possible in principle to monitor for the likely presence of this failure mode in software (manifesting as a steady increase in current provided to the solenoid, with concurrent decrease in VT delivered, over a period of hours). Further testing would be required to investigate the lifetime of the electro-mechanical parts.

### Pre-clinical testing

OxVent was able to deliver a set VT with PEEP values ranging between 5 and 20 cmH_2_O. The loss of PEEP and ongoing expiratory flow were isolated to a leak from the PEEP valve specifically during these tests caused by minor damage likely occurred during transport in the research expedition. However, in standard conditions, the PEEP valve operated as expected (see section Independent evaluation). Qualitatively, the OxVent was easy to set-up and use. Further work is needed to determine the ventilator's capability to support spontaneous breathing in patients.

### Independent evaluation

Independent evaluation noted the device's low oxygen consumption, and that ASV mode *“works well referenced to PEEP”*. With regards to usability, the device was described as “*compact and lightweight; reasonably easy to clean”*, and though the design is *“wholly unfamiliar [for] intensivists”*, with a *“30* min *period of intense training a non-clinical student, with knowledge of the basics of mechanical ventilation, could set it to start-up settings and start ventilation”*. The potential for repeated use of single-use patient-facing components (such as hoses and HME filters) was an area of concern and would need to be mitigated via training.^18^

In summary, our study demonstrates that the OxVent mechanical ventilator design, based on components outside the standard ventilator supply chain, operates to standards that largely satisfy the MHRA RMVS specifications. With further design work (notably, making it easier to replace worn-out parts and adding software to monitor against degradation), the long-term durability of the system could be further improved beyond the specified two-week lifetime. This design has the potential to be manufactured at large scale and meet demand when a rapid increase in mechanical ventilation capacity is required.

## Declaration of interests

FFo reports grants from the National Institute for Health Research (UK), the National Institute of Academic Anaesthesia, and the Wellcome/EPSRC Centre for Medical Engineering. AF, FFo, SO and MT are volunteering directors of OxVent, a joint-venture social enterprise for mechanical ventilation between Oxford University and King's College London. TD is on the advisory board of OxVent. AAC-P, AF, FFo, MT, PG, SO and TD have shares in OxVent Ltd. AH and CVF are part-time employees of OxVent Ltd.
